# Ependymoma: Evaluation and Management Updates

**DOI:** 10.1007/s11912-022-01260-w

**Published:** 2022-04-06

**Authors:** Roberta Rudà, Francesco Bruno, Alessia Pellerino, Riccardo Soffietti

**Affiliations:** Division of Neuro-Oncology, Department of Neuroscience “Rita Levi Montalcini”, University of Turin, Via Cherasco 15, 10126 Turin, Italy

**Keywords:** Supratentorial ependymomas, Posterior fossa ependymomas, Spinal cord ependymomas, ZFTA fusion-positive ependymoma, YAP-1 fusion-positive ependymoma, Posterior fossa A, Posterior fossa B, MYCN-amplified spinal ependymoma, Myxopapillary ependymoma, Surgery, Conformal radiotherapy, Stereotactic radiotherapy, Proton therapy, Chemotherapy

## Abstract

**Purpose of Review:**

To review state of art and relevant advances in the molecular genetics and management of ependymomas of children and adults.

**Recent Findings:**

Ependymomas may occur either in the brain or in the spinal cord. Compared with intracranial ependymomas, spinal ependymomas are less frequent and exhibit a better prognosis. The new WHO classification of CNS tumors of 2021 has subdivided ependymomas into different histomolecular subgroups with different outcome. The majority of studies have shown a major impact of extent of resection; thus, a complete resection must be performed, whenever possible, at first surgery or at reoperation. Conformal radiotherapy is recommended for grade 3 or incompletely resected grade II tumors. Proton therapy is increasingly employed especially in children to reduce the risk of neurocognitive and endocrine sequelae. Craniospinal irradiation is reserved for metastatic disease. Chemotherapy is not useful as primary treatment and is commonly employed as salvage treatment for patients failing surgery and radiotherapy.

**Summary:**

Standard treatments are still the mainstay of treatment: the discovery of new druggable pathways will hopefully increase the therapeutic armamentarium in the near future.

## Introduction

Ependymomas are neuroepithelial tumors that may arise from the ependymal cells of the cerebral ventricles, the central canal of the spinal cord, or cortical rests. According to the Central Brain Tumor Registry of the USA, the annual incidence of ependymomas ranges from 0.29 to 0.6 per 100,000 persons [1]. These tumors account for 1.6–1.8% of all primary CNS tumors: in children they are proportionally more common being 5.2%, while in adults are around 4%. Males are slightly more affected than females (1.3:1). Tumor location is largely dependent on patient age, with approximately 90% of pediatric ependymomas occurring intracranially, and 65% of adult tumors occurring in the spinal cord [2].

Given the low incidence, the literature regarding intracranial ependymomas in adults is sparse, as most series combine pediatric and adult ependymomas, grade II and grade III tumors, are retrospective, include limited numbers of patients, and span several decades in which diagnostic and therapeutic modalities have changed. Conversely, more information is available in ependymomas of children, and commonly therapeutic strategies in adults follow those in children [3].

In recent years, the molecular changes in ependymoma have undergone extensive analysis: the studies have provided new insight to define the origin of the ependymoma stem cell and generated new prognostic markers; however, this knowledge has not translated into the identification of druggable targets [4–6, 7•].

A recent molecular classification has distinguished 9 subgroups of ependymomas that appear to reflect more precisely than histology alone the biological, clinical, and histopathological heterogeneity across the major anatomical compartments, age groups, and tumor grades and have represented the basis for the update of World Health Organization (WHO) classification from 2016 to 2021 [8–10, 11••].

## The New WHO Classification of 2021

According to the WHO classification of CNS tumors of 2021 [11••], when possible, ependymomas should be classified according to a combination of histological and molecular features across three tumor locations (supratentorial, infratentorial and spinal). One group at each anatomical site consists of tumors with the morphological features of subependymoma (WHO grade 1), that is often asymptomatic, discovered only incidentally on neuroimaging and carries an excellent prognosis.

In the supratentorial compartment, there are two other groups with different molecular features, clinical characteristics and outcome, i.e., the supratentorial ependymoma ZFTA fusion-positive and the supratentorial ependymoma YAP1 fusion-positive. The first one accounts for the majority of supratentorial ependymomas and may occur both in children and adults. The second one is uncommon and restricted to young children. In terms of prognosis, a retrospective study reported a poorer survival for ST-RELA [8], while in trial-based analyses of pediatric cohorts, RELA-fusion status was not found to be prognostic [12•, 13, 14•]. The ST-YAP1 fusion-positive ependymoma seems to carry a favorable prognosis [13, 15•]. Unlike ST ependymomas, posterior fossa ependymomas lack recurrent fusions, while methylation profiling has identified two main subgroups, i.e., posterior fossa A (PFA) and B (PFB). Moreover, the level of histone H3K27-trimethylation is high in PFB but low in PFA tumors. Gain of 1q has been shown across multiple studies to be a highly prognostic independent marker of a poor outcome in PFA ependymomas [12•, 14•, 15•] but not in PFB ependymomas [16]. In general, additional molecular heterogeneity within PFA ependymomas is being discovered [17•]. PFA ependymomas occur mainly in infants and children, while PFB ependymomas arise mainly in adolescents and adults. PFA ependymomas have a poor prognosis compared with that of PFB ependymomas [8].

In the spinal cord, classical ependymomas (WHO grade 2 and 3) and myxopapillary ependymomas (WHO grade 2) predominate among adults and thus far are not stratified molecularly: they are often relatively indolent tumors, while a rare and highly aggressive subgroup with a propensity to disseminate, affecting young adults and adolescents, the spinal ependymoma, MYCN-amplified, has been recently identified [18, 19•]. Due to the unfavorable prognosis, now, this tumor type should be distinguished from the other two spinal subgroups to guide clinical management.

Overall, despite the classification of ependymomas moving towards a molecular diagnosis, the WHO grading system still holds prognostic value, especially the distinction of grades 2 and 3 in intracranial ependymomas, and can help guide treatment decisions [20]. This is particularly true for only histologically defined tumors, when a canonical molecular signature is absent, when the molecular definition is uncertain due to the presence of mutations of unknown significance, or in places where molecular testing is not available.

## Neuroimaging for Diagnosis and Monitoring

MRI with contrast enhancement is the modality of choice for diagnosing ependymal tumors [21, 22]. CT can better depict calcifications, which are most commonly observed in subependymomas.

Intracranial ependymomas commonly appear as well-circumscribed mass lesions and have a heterogeneous appearance on T1-, T2-, and post-contrast magnetic resonance imaging (MRI), displaying varying degrees of contrast enhancement. Advanced imaging modalities may assist in diagnosis. Diffusion-weighted imaging may be useful for differentiating pilocytic astrocytomas, medulloblastomas, and ependymomas in the posterior fossa [23]. MR spectroscopy reveals elevated choline and reduced N-acetylaspartate levels [6]. Perfusion MRI may display elevated cerebral blood volume values and have some prognostic value [24].

Spinal cord ependymomas display more distinct borders than diffuse astrocytomas [25]. Cyst formation and T2 hypointensity of the cyst wall due to blood products (“hemosiderin cap”) are suggestive of ependymoma, and an associated syringomyelia is common.

Myxopapillary ependymoma (MPE) is typically located in the conus medullaris, cauda equina, and filum terminale region.

Surveillance with MRI may detect recurrences in asymptomatic patients for earlier salvage therapies, resulting in superior 3-year progression free survival (PFS), but the impact on overall survival (OS) is still limited [26•]. It is still not clear how often and for how long surveillance is needed [27]; however, due to the possibility of late recurrences, long-term follow-up is standard.

## Management of Newly Diagnosed Intracranial Ependymomas in Children

Gross total resection is the mainstay of treatment with an OS of at least 70% at 5 years [7•, 14•, 28, 29]. The possibility of avoiding adjuvant radiotherapy has been explored in the ACNS0121 trial [12•]: 11 patients with completely resected supratentorial tumors of WHO grade 2 were observed with MRI, achieving a 5-year PFS of 61.4% and OS of 100%. The likelihood of a GTR is greater for supratentorial tumors than for infratentorial ones. Tumors arising from the floor and the lateral portion of the IV ventricle have a higher risk of postoperative deficits as compared to those arising from the roof, and subtotal resections are more often performed with the consequence of a lower OS. Due to the prognostic value of GTR, a second-look surgery is increasingly employed in case the first resection did not achieve a GTR, commonly when the surgical procedure was performed in an emergency situation or in a non-skilled institution [30, 31].

The benefit of postoperative radiotherapy for incompletely resected ependymomas in terms of local control and overall survival rates is clear [12•, 29, 32]. The postoperative radiotherapy of 59.4 Gy (1.8 Gy/fraction) in grade 3 and grade 2 incompletely resected tumors is recommended [32] for children older than 3 years, while for children as young as 18 months or children with altered neurological status, the doses can be lowered to 54 Gy [33]. This can be true even for children between 12 and 18 months. A recent retrospective study on 206 patients reported that the main pattern of relapse is within the radiation fields even at 59.4 Gy [34]. A hypofractionated stereotactic boost in addition to conventional radiotherapy has been proposed to increase the efficacy [35]. A prospective study showed that hyperfractionated radiotherapy is safe but provides no outcome benefit compared with a standard fractionated regimen [36].

The toxicity of radiotherapy in younger children is of concern, and intensity-modulated radiation therapy is employed to limit late sequelae [37]. Proton therapy could be an alternative to conventional photon radiotherapy for toxicity reduction [38]. Reducing neurocognitive dysfunction, hearing loss, and neuroendocrine deficits is an important priority, especially for young children. Proton therapy may offer disease control commensurate with modern photon therapy [39, 40•]. A recent study evaluating proton therapy [40•] reported a 7-year local control, progression-free survival, and overall survival rates of 77%, 64%, and 82%, which are comparable to values reported by a parallel study with photon therapy (72%, 60%, and 79%) [12•]. The rates of symptomatic brain stem toxicity (4%), vasculopathy (1.8%), hearing loss (5%), and hormone deficiency (12%) with proton therapy appear slightly lower than those observed with conventional radiotherapy.

The role of chemotherapy in children remains unproven despite intensive investigation [41]. As there is reluctance to deliver radiation to very young children, postoperative chemotherapy is frequently proposed, while in older children, chemotherapy is delivered as an adjunct to radiotherapy. In prospective clinical trials, postoperative chemotherapy using various combinations of etoposide, vincristine, cyclophosphamide, platinum derivatives, and high-dose methotrexate demonstrated a 40 to 50% response rate [37, 42–44].

Intensified chemotherapy schedules have been suggested in infants [43], especially for those with supratentorial tumors [45]. In contrast, the use of immediate postoperative high-dose conformal radiotherapy in children under the age of 3 years is being investigated, but long-term follow-up for toxicity is still pending. Overall, radiotherapy deferral strategies by using chemotherapy for children aged more than 12 months have been abandoned also due to inadequate tumor control by chemotherapy.

Thus far, no targeted therapies are available in ependymomas. A recent preclinical study has suggested FGFR inhibition as a novel approach to target aggressive ependymomas [46].

## Management of Newly Diagnosed Intracranial Ependymomas in Adults

Surgery is the first step of standard treatment. In the majority of studies, the extent of resection has emerged as one of the most significant predictors of outcome [2, 47–52, 53•]. In a retrospective series of WHO grade 2 ependymomas in adults [51], gross total resection (GTR) and infratentorial location were associated with a longer OS. GTR and tumor location were also independent factors predicting progression-free survival (PFS). Conversely, incomplete resection has an increased risk of tumor recurrence and CSF dissemination. However, in posterior fossa tumors, encasement of the cranial nerves and brainstem vasculature might limit extent of tumor resection [54].

In the past, patients with ependymoma often received craniospinal irradiation. However, numerous studies demonstrated the efficacy of local fields in the treatment of ependymoma, achieving good local control with low risk of spinal dissemination [55, 56•]. In adults, there is agreement that postoperative radiotherapy should be included in the standard of care for patients with anaplastic ependymoma (WHO grade 3) and for patients with ependymomas (WHO grade 2) after an incomplete resection [49, 57]. Conversely, the role of postoperative radiotherapy in patients with ependymoma WHO grade 2 undergoing GTR remains controversial [58].

Chemotherapy has no role in the adjuvant setting of newly diagnosed intracranial ependymomas of the adult.

## Tailoring Treatment to Molecular Subgroups

Thus far, there are no data from prospective clinical studies tailored to the different molecular subgroups. Some hypotheses, generated in retrospective translational studies, need validation in clinical trials. Among supratentorial ependymomas, the subgroup with YAP1 fusion, which displays an excellent prognosis, could be observed without further therapy after resection. The same could be true for posterior fossa B tumors, in which also a de-escalation of RT could be considered. Conversely, posterior fossa A ependymomas are aggressive tumors, and the role of adjuvant radiotherapy after complete resection should be considered. Moreover, a recent study showed that tumor cells with increased EZHIP expression, which is considered as an important oncogenic driver in PFA, suppress DNA repair and respond to PARP inhibitors, especially when associated with radiotherapy [59]. Whether supratentorial ependymomas with RELA fusion need more intensive treatment is unknown and should be investigated.

## Management of Recurrent Intracranial Ependymomas

Primary therapies for intracranial ependymomas, especially in children, are insufficient to prevent tumor recurrence. The majority of recurrences develop within the first 2 years of diagnosis; however, some of them occur many years later. Overall, there is no consensus on the best approach. Unfortunately, there have been no recent novel treatments on the horizon, apart from CAR-T cell therapy, which is still at the beginning [60•].

The extent of resection at recurrence appears important in improving the outcome [61–63].

A recent study in children and adolescents [64•] has reported that gross- and near-total resection were achieved in 64% of patients and were associated with an improved 5-year survival (OS) of 48.7% vs 5.3% in less than gross total or near-total resection.

There is also evidence that reirradiation may be beneficial in both adults and children at time of recurrence, using either conventionally fractionated irradiation or stereotactic irradiation or proton therapy [65–69]. The efficacy is more evident following incomplete resection [64•], but the duration of control could be limited in time [63, 70•].

Phase II studies in children with relapsing ependymomas have reported low response rates with either standard [71] or high-dose chemotherapy [72]. Metronomic therapies may yield long-term control [73]. Oral etoposide [74] or temozolomide (TMZ) [75] may yield some responses. Bevacizumab, in association with either irinotecan [76] or lapatinib [77], does not seem to be active. Targeted agents, such as erlotinib and sunitinib [74, 78], did not show any activity in an unselected series of patients.

Chemotherapy for recurrent ependymoma in adults is considered only when local options (surgery and radiotherapy) have been exhausted [3, 65, 79].

Similar to diffuse gliomas, TMZ has been used for the treatment of adult patients with ependymoma. A retrospective study conducted by Rudà et al. included 18 patients with recurrent WHO grade 2 and 3 intracranial ependymomas failing re-operation and/or re-irradiation, suggested an efficacy of TMZ in the standard schedule in terms of response (22% complete + partial) and survival (PFS 9.69 months and OS 30.55 months) [80]. Responses were observed in chemotherapy-naïve patients and tended to be delayed. Conversely, in another retrospective study conducted by Chamberlin et al. of patients with WHO grade 2 intracranial ependymomas refractory to first-line chemotherapy with platinum compounds [81], TMZ in the standard schedule had a limited efficacy with a response rate of 4%, a PFS of 2 months, and OS of 3 months. One must take into account that in the cohort of Chamberlain [81], patients were heavily pretreated, while the majority of patients in the cohort of Rudà [80] were chemo-naïve, thus receiving TMZ in an earlier phase of the disease. Temozolomide has also been used in combination with lapatinib in a single-arm phase II study in patients with recurrent intracranial and spinal ependymomas [82]. Lapatinib targets the epidermal growth factor receptors (ErbB1 and ErbB2), which may be expressed by ependymoma cells. Fifty patients were enrolled with a median PFS of 45 weeks for patients with WHO grade 2 tumors and 25.3 weeks for patients with WHO grade 3 ependymomas. Responses to treatment correlated with higher ErbB2 mRNA expression in tumor tissue. The modest activity of TMZ against ependymoma cells has been suggested to be due to the lack of O6-methylguanine-DNA-methyltransferase (MGMT) promoter methylation [83, 84]; however, even when present, MGMT promoter methylation does not correlate with response to TMZ [80].

Platinum-based regimens seem superior over nitrosourea-based regimens [85]. A retrospective series reported higher response rates in patients with progressive or recurrent ependymoma treated with cisplatin compared with non-platinum regimens, but no difference in terms of PFS and OS was observed [86]. The anti-angiogenic agent bevacizumab has been employed in 8 patients with recurrent WHO grade 2 or 3 adult intracranial ependymoma with a median PFS of 6.4 months and OS of 9.4 months [87].

Table 1 summarizes the ongoing clinical trials in recurrent ependymomas.

**Table 1:** Ongoing clinical trials in recurrent / progressive ependymomas

NCT number	Type of study	No. of patients	Type of treatment	Estimated study completion
NCT02155920	Phase 2	11 patients with recurrent or progressive grade 2–3 ependymomas	Everolimus 4.5 mg/m^2^/dose once daily	December 30, 2021
NCT04958486	Early phase 1	10 children and adults with recurrent or residual posterior fossa ependymoma	5-azacytidine and trastuzumab infusions into the fourth ventricle or resection cavity	July 5, 2023
NCT01795313	Phase 1	24 patients aged 1–21 years with recurrent ependymomas (any grade)	HLA-A2 restricted tumor antigen peptide vaccine plus imiquimod	December 31, 2023
NCT03572530	Phase 1	9 patients aged 1–80 years with recurrent ependymomas	5-azacytidine infusions into the fourth ventricle Arm 1: 3 times per week Arm 2: 2 times per week Arm 3: 1 time for week	July 1, 2022
NCT02774421	Phase 1	33 patients aged 1–21 years with recurrent posterior fossa ependymomas	intrathecal trastuzumab in combination plus subcutaneous granulocyte–macrophage colony-stimulating factor	March 1, 2022
NCT03033992	Not applicable	25 patients with recurrent or progressive supratentorial malignant gliomas and ependymomas	Tumor treating fields device	September 30, 2022
NCT04661384	Phase 1	Leptomeningeal metastases from ependymomas	IL13Ralpha2-CAR T cells	December 15, 2023
NCT02359565	Phase 1	100 children with recurrent or progressive diffuse intrinsic pontine glioma, high-grade gliomas, ependymomas, medulloblastomas or hypermutated brain tumors	Pembrolizumab every 21 days	December 31, 2024

## Management of Spinal Cord Ependymomas

Spinal ependymoma (grade 2 or 3) has a better outcome than spinal astrocytoma. GTR offers the best prognosis [88•]. Advances in microsurgical techniques allow en bloc GTR over piecemeal subtotal resection (STR) as standard of care for spinal ependymomas with good functional results. Resection should be considered at an early stage of the disease as functional outcome is related to small tumor size and good neurological status at the time of surgery [89•, 90]. When GTR is not feasible, postoperative conformal radiotherapy is employed. A review of the literature on 348 patients with WHO grades 2 and 3 spinal ependymomas [91] has shown that the extent of resection and tumor grade were independent prognostic factors for OS and PFS, and radiotherapy prolonged PFS in patients receiving STR. Median PFS was 48 months in patients treated with STR alone and 96 months for patients treated with STR followed by radiotherapy. Studies suggest doses of ≥ 50 Gy [91].

Spinal ependymoma with MYCN amplification has a poor prognosis, but it is unknown what is the best therapeutic management for this subtype. Strategies for MYCN inhibition are being investigated, including vaccination [92].

Continuous oral etoposide is well tolerated and may be active in recurrent intramedullary ependymoma; also, bevacizumab can provide clinical benefit in some patients [93].

Large retrospective series on MPE are available. A multi-institutional series of 183 patients [94] showed a 10-year OS of 92.4% and a 5- and 10-year PFS of 69.5% and 61.2%. Recurrence was local in 84% of patients, while leptomeningeal spread occurred in 9.3% of patients. An OS at 10 years exceeding 90% has been recently confirmed in an analysis by the Surveillance, Epidemiology, and End Results (SEER) program of 773 patients [95].

Surgical resection is the mainstay of treatment for MPE, and the extent of resection is the most important prognostic factor [94, 96, 97, 98•]. However, due to a close adhesion to the nerve roots and production of a myxoid matrix, a safe GTR of MPE may be challenging: in fact, the rate of GTR in the literature ranges from 53 to 75% [98•, 99•]. A systematic review of the literature [100] found that the recurrence rate after GTR (15.5%) was significantly lower than that after STR (32.6%), while being higher in pediatric patients as compared to adults (40.5% vs 23.4%). The role of radiotherapy for MPE in adults is still controversial. Adjuvant radiotherapy was not associated with a decrease in the recurrence rates in the review of Feldman et al. (2013), while other series reported a superiority for combined surgery and postoperative radiotherapy over surgery alone [96, 99•, 101]. In particular, the experience of MD Anderson Cancer Center [96] showed that the addition of postoperative radiotherapy to surgery was associated with significantly longer 10-year PFS rates (75% for the combination vs 37% for surgery alone).

MPE is very rare in children. Pediatric patients frequently present with disseminated tumor and/or develop recurrent or progressive disease following treatments [102], though the OS at 5 and 10 years in the SEER database is estimated at 97% and 95%, respectively [103]. Recent small studies suggested a better local control after surgery and postoperative radiotherapy [104, 105].

In general, the definitive role of radiotherapy still needs further studies. The irregular shape, contact with surrounding nerve roots, and production of a myxoid matrix, particularly in the filum terminale, can make GTR particularly challenging with risks of postoperative neurological disability. A strong correlation between capsular violation at surgery and recurrence has been found [97]. Considering the non-negligible risk of recurrence and spinal dissemination of MPE, radiotherapy should be recommended for patients undergoing largely incomplete resections, even if there is some risk of post-irradiation radiculopathy in long-term survivors.

## Conclusions

Recent translational studies show that most ependymomas have a similar appearance using conventional microscopy but vary widely in the genetic alterations that likely led to tumorigenesis and subsequent tumor biology.

Most studies demonstrate a major impact of GTR on survival outcomes, despite small patient numbers limiting reliable multivariate analyses. For incompletely resected intracranial grade 2 tumors, postoperative radiotherapy is recommended. The predominant pattern of relapse is local; thus, it is important for all patients to undergo complete resection when possible and a “second surgery” for those with initial incomplete resection. The benefit of radiotherapy could be enhanced by dose escalation using modern techniques such as proton therapy. CSI could be useful in patients with leptomeningeal spread. A better knowledge of molecular pathways of tumor progression is needed to develop novel targeted agents. Multi-institutional and international studies are necessary to tailor the different therapeutic options to the different molecular subgroups.
